# Genetic manipulations of nonmodel gut microbes

**DOI:** 10.1002/imt2.216

**Published:** 2024-06-23

**Authors:** Wen‐Bing Jin, Chun‐Jun Guo

**Affiliations:** ^1^ Jill Roberts Institute for Research in Inflammatory Bowel Disease, Weill Cornell Medicine Cornell University New York New York USA; ^2^ Friedman Center for Nutrition and Inflammation, Weill Cornell Medicine Cornell University New York New York USA; ^3^ Joan and Sanford I. Weill Department of Medicine, Gastroenterology and Hepatology Division, Weill Cornell Medicine Cornell University New York New York USA; ^4^ Department of Microbiology and Immunology, Weill Cornell Medicine Cornell University New York New York USA; ^5^ Immunology and Microbial Pathogenesis Program, Weill Cornell Graduate School of Medical Sciences, Weill Cornell Medicine Cornell University New York New York USA

**Keywords:** genetic manipulation strategies, human gut microbiota, nonmodel gut *Bacteroidia*, nonmodel gut *Clostridia*

## Abstract

Hundreds of microbiota gene expressions are significantly different between healthy and diseased humans. The “bottleneck” preventing a mechanistic dissection of how they affect host biology/disease is that many genes are encoded by nonmodel gut commensals and not genetically manipulatable. Approaches to efficiently identify their gene transfer methodologies and build their gene manipulation tools would enable mechanistic dissections of their impact on host physiology. This paper will introduce a step‐by‐step protocol to identify gene transfer conditions and build the gene manipulation tools for nonmodel gut microbes, focusing on Gram‐negative *Bacteroidia* and Gram‐positive *Clostridia* organisms. This protocol enables us to identify gene transfer methods and develop gene manipulation tools without prior knowledge of their genome sequences, by targeting bacterial 16s ribosomal RNAs or expanding their compatible replication origins combined with clustered regularly interspaced short palindromic repeats machinery. Such an efficient and generalizable approach will facilitate functional studies that causally connect gut microbiota genes to host diseases.

## Highlights

- A protocol introducing a step‐by‐step genetic manipulation method would facilitate the investigation of those functional genes encoded by nonmodel gut commensals.
- The gene‐editing tools could be established in nonmodel gut *Bacteroidia* and *Clostridia* without prior knowledge of their genome information.
- The genetic manipulation pipeline may serve as a high‐throughput genetics screening and manipulating platform for human gut microbes.

## Graphical abstract

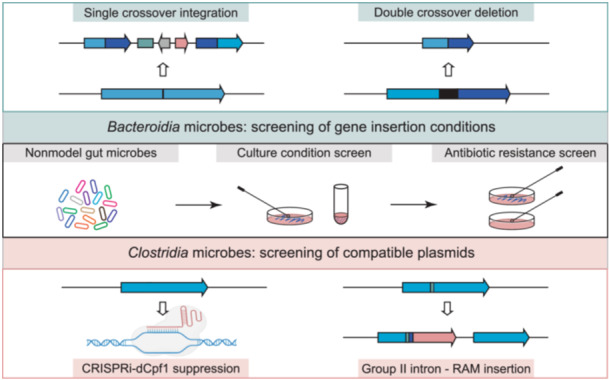

## INTRODUCTION

The gut microbiota impacts human biology in many ways. Multiomics studies revealed many microbiota genes whose expressions significantly differ between healthy and diseased humans [1, 2, 3, 4, 5, 6]. However, unraveling the causal molecular mechanisms underlying microbiota gene–host biology interactions remains challenging, mainly due to limited approaches to precisely manipulating these disease/biology‐associated microbes and their metabolic genes.

Developing genetic manipulation tools for nonmodel gut microbes is necessary because: (1) Previous studies have revealed that host diseases are significantly associated with microbiota genes [1, 2, 3, 4, 5, 6]. Those genes are mostly expressed in nonmodel gut microbes that are not genetically tractable. Establishing genetic tools will be the first step to manipulating their gene expression within the host, and further to study their impact on human diseases. (2) Human biology is profoundly regulated by gut microbiota, yet the knowledge about which gut microbes and genes play an essential role remains largely unstudied. Genetic manipulation tools will facilitate the functional studies of physiology interactions between gut microbiota and host.

Here, we reported a step‐by‐step protocol to build the genetic manipulation strategies for nonmodel gut *Bacteroidia* and *Clostridia* microbes, whose abundances dominate healthy human guts [7, 8]. By targeting bacterial 16s ribosomal RNAs (rRNAs) or expanding their compatible replication origins combined with clustered regularly interspaced short palindromic repeats (CRISPR) machinery, this pipeline enables us to identify exogenous genomic DNA transfer methodologies and develop genetic tools without prior knowledge of the genome sequence of those nonmodel gut microbes.

## GENETIC MANIPULATION STRATEGIES

### Identifying gene transfer methods for *Bacteroidia* microbes and building their gene insertion tools

*Escherichia coli* (*E. coli*) conjugation was used to introduce the exogenous DNA into the recipient microbes, as the method has been proven effective in some *Bacteroides* and *Clostridium* [9, 10, 11, 12, 13, 14, 15, 16, 17, 18, 19, 20]. Culture conditions of strains on agar plates and in liquid broth were first screened and then these microbes were tested against a collection of antibiotics to (1) find an antibiotic they are susceptible to, so its resistance gene will be used as a universal selective marker, and (2) identify an antibiotic the recipient microbe is resistant to but not the donor *E. coli*, so it can be used as an additive to suppress *E. coli* growth after conjugation.

The *Bacteroidia* are Gram‐negative obligate anaerobes that uptake exogenous DNA and have efficient homologous recombination (HR) [16]. To establish a generalizable approach for the genetic manipulation of *Bacteroidia* (*Bacteroides* and *Prevotella*) microbes, conserved bacterial 16s rRNA gene, whose sequence has been widely used to assess microbiome diversity and construct bacterial phylogeny, was selected as a universal target. To do this, a synthesized chimeric 16s (chi‐16s) sequence, with high homology to the *Bacteroidia* 16s rRNA genes, was assembled with a suicide conjugation vector, and transported into recipient *Bacteroidia* microbes. Those transconjugants whose 16s rRNA loci have been inserted by the suicide vector will be genetically tractable.

#### Materials and devices

Primer star DNA polymerase (Takara, Cat# R045), Blue sapphire DNA polymerase (Takara, Cat# RR350), Plasmid Midiprep Kit (Zymo Research, Cat# D4201), DNA Clean and Concentrator (Zymo Research, Cat# D4003), Tryptic Soy Agar (BD, Cat# 236950), Brain Heart Infusion Agar (BD, Cat# 241830), Columbia Blood Agar (CBA) (BD, Cat# 279240), Horse blood (Hemostat Laboratories, Cat# 637291), Luria–Bertani (LB) broth (BD, Cat# BP1426), glycerol (Fisher Bioreagents, Cat# BP229), the vacuum‐pumping system, phosphate‐buffered saline (PBS) (Gibco, Cat# 10010‐031), centrifuge, polymerase chain reaction (PCR) amplifier, d‐cycloserine (D) (TCI, Cat# C1189), gentamicin (G) (GoldBio, Cat# G‐400‐25), kanamycin (K) (GoldBio, Cat# K‐120‐25), carbenicillin (GoldBio, Cat# C‐103‐25), thiamphenicol (Thiam) (Acros Organics, Cat# 455450250), anaerobic chamber, aerobic incubator, electroporation system, Thermo Scientific Nanodrop 2000, Gibson Assembly Cloning Kit (NEB, Cat# E5510S), Quick DNA fungal/bacterial kit (Zymo Research, Cat# D6005), 50 mL Tube Top Vacuum Filter System (0.22 mm) (Corning Life Sciences, Cat. #430320), and ultralow temperature freezer.

#### Screening of culture conditions

The culture of Gram‐negative *Bacteroidia* strains was incubated in an anaerobic chamber at 37°C under an atmosphere of 5% carbon dioxide (CO_2_), 7.5% hydrogen (H_2_), and 87.5% nitrogen (N_2_). The agar plates were left in the anaerobic chamber for at least one overnight before use. The liquid medium was left in the chamber with a loosened cap for at least 48 h before inoculation.

**Figure 1:**
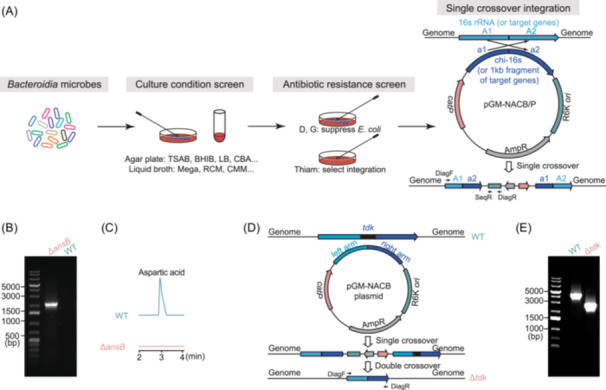
Workflow for identifying gene transfer methods for *Bacteroidia* microbes and developing genetic manipulation tools for *Bacteroidia* microbes via single crossover insertion and double crossover deletion. (A) Workflow for identifying gene transfer methods for *Bacteroidia* microbes. (B, C) Genetic manipulation of asparaginase gene (*ansB*) in *Prevotella bivia* DSM 20514 via single crossover integration. (B) Diagnostic polymerase chain reaction (PCR) showed that the mutant strain (Δ*ansB*) had the PCR product of ~2 kb, while the wide type (WT) strain had no band. (C) Liquid chromatography–mass spectrometry trace showed that the mutant strain (Δ*ansB*) lost the ability to convert substrate asparagine to aspartic acid. (D, E) Genetic manipulation of thymidine kinase gene (*tdk*) in *Bacteroides* sp. 1_1_30 via double crossover deletion. (D) Schematic view of knocking out gene *tdk* in gut bacteria *Bacteroides* sp. 1_1_30 using a double crossover recyclable marker system. (E) Diagnostic PCR showed that the mutant strain (Δ*tdk*) had the expected shorter PCR product compared with the WT strain. BHIB, Brain Heart Infusion Agar + Horse blood; CBA, Columbia Blood Agar; chi‐16s, chimeric 16s; CMM, Chopped Meat Medium; D, d‐cycloserine; diagF, diagnostic forward primer; diagR, diagnostic reverse primer; *E. coli*, *Escherichia coli*; G, gentamicin; LB, Luria–Bertani; RCM, Reinforced Clostridial Medium; rRNA, ribosomal RNA; seqR, sequencing primer (reverse); Thiam, thiamphenicol; TSAB, Tryptic Soy Agar + Horse blood.

Strains were restreaked (from original glycerol stock) onto pre‐reduced agar plates (such as Tryptic Soy Agar + 5% blood [TSAB] plates, Brain Heart Infusion Agar + 5% blood [BHIB] plates, or CBA plates). Then, those that can grow on agar plates were subcultured into pre‐reduced liquid medium: Mega, Chopped Meat Medium (CMM), and Reinforced Clostridial Medium (RCM, BD, Cat# 218081). Strains that can grow in any of the four liquid cultures were subjected to the antibiotics test (Figure 1A).

*Keynotes*: For the screening of the culture conditions of liquid medium, *Bacteroidia* strains need to be first recovered on agar plates to ensure *Bacteroidia* strains are activated, instead of inoculating *Bacteroidia* strains into liquid medium from the frozen glycerol stock directly.

*Potential issues and solutions*: If the *Bacteroidia* strains of interest cannot grow on the common agar plates or in the liquid medium listed above, other specific plates or liquid mediums that favor the growth of the target strains need to be used to screen the culture conditions.

#### Antibiotic test

To find the antibiotic that suppresses the growth of conjugation donor *E. coli* S17, the *Bacteroidia* (*Bacteroides* and *Prevotella*) microbes were restreaked on agar plates supplemented with 200 µg/mL gentamicin (G) or 250 µg/mL d‐cycloserine (D). The tested *Bacteroidia* microbes are expected to grow on plates with either gentamicin or d‐cycloserine. Most of the *Bacteroidia* strains we tested so far are sensitive to thiamphenicol (Thiam), so the thiamphenicol‐resistant gene (*catP*) can be used as a universal marker to select transconjugants whose genome was integrated by the suicide vector. The minimum inhibitory concentrations (MICs) of thiamphenicol of the *Bacteroidia* microbes were tested on agar plates containing thiamphenicol at different concentrations (Figure 1A).

*Keynotes*: Antibiotics need to be added into the agar medium before the agar plates are poured and solidified; agar plates supplemented with antibiotics only on the surface of the plates may cause misleading antibiotic test results.

*Potential issues and solutions*: If the *Bacteroidia* strains of interest are not resistant against gentamicin or d‐cycloserine, other specific antibiotics that can suppress the growth of *E. coli* S17 can be used for the test. Likewise, if the *Bacteroidia* strains of interest are resistant against thiamphenicol, the antibiotic marker on the suicide vector can be replaced by other markers, whose corresponding antibiotics can suppress the growth of the *Bacteroidia* strains.

#### Vector assembly

By assembling the synthesized conserved ~1 kb chi‐16s rRNA sequence (chi‐16s) for *Bacteroidia* with the suicide vector pExchange [21], plasmids pGM‐NACB (targeting 16s rRNA gene of *Bacteroides*, GM: genetic manipulation, N: no gram‐positive replication origin, A: R6K gram‐negative replication origin, C: *catP* antibiotic marker, B: *Bacteroides*) and pGM‐NACP (targeting 16s rRNA gene of *Prevotella*, P: *Prevotella*) were generated [2] (Figure 1A). Likewise, to target other specific genes in *Bacteroidia* strains, an ~1 kb fragment of the target gene could be amplified by PCR and assembled with the backbone amplified from the suicide vector pGM‐NACB/P to get the plasmid for mutating the target gene via insertion–deletion (Figure 1A).

*Keynotes*: In the case the size of the target gene is smaller than 1 kb, a ~0.5 kb fragment of the target gene is also functional for the single crossover, the key is that the fragment has to be an incomplete fraction (both upstream and downstream) of the target gene.

#### Introduction of suicide vectors into the *Bacteroidia* microbes

The suicide vectors pGM‐NACB/P were introduced into the target *Bacteroides*/*Prevotella* using *E. coli* conjugation following the previously published protocol [21]. A single colony of the target *Bacteroidia* microbe was inoculated in 3 mL liquid broth and cultured in an anaerobic chamber at 37°C. The *E. coli* S17 harboring the pGM‐NACB/P vector was inoculated in the LB broth supplemented with carbenicillin (100 µg/mL) and grown at 37°C with aerobic shaking at 220 rpm. After ~12–16 h, when the OD_600_ of *E. coli* S17 reached 0.8–1.0, 6 mL of *E. coli* S17 culture was centrifuged at 1500*g* for 2 min. The supernatant was discarded, and the cell pellet was washed twice with 3 mL PBS buffer (pH = 7.4). The washed *E. coli* S17 cell pellet was resuspended in a 3 mL overnight culture of the target *Bacteroidia* strain and gently mixed by pipetting. The mixture was filtered through a 0.2 µm filter. The filtered liquid was discarded. The filter with the mixture of donor and recipient cells was placed onto the surface of a pre‐reduced agar plate (cell surface facing down). The plate was incubated aerobically in a 37°C incubator.

After incubation aerobically at 37°C for 24 h, the filter was soaked in 2 mL pre‐reduced liquid broth. The cell on the filter was resuspended into the broth by gentle vortexing. The mixture was then transferred into the anaerobic chamber, and 100 µL (or serial diluted suspension) was plated onto a pre‐reduced agar plate with 200 µg/mL gentamicin + 15 µg/mL thiamphenicol (or MICs). Colonies of the target strain typically appeared after 36–48 h. Four colonies were picked and restreaked on a pre‐reduced agar plate with 200 µg/mL gentamicin + 15 µg/mL thiamphenicol (or MICs) to isolate single colonies.

*Keynotes*: The target *Bacteroidia* microbes are cultured in the anaerobic chamber overnight (~12 h). Do not culture the *Bacteroidia* strains for too long before conjugation, it will lead to the lysis of the strains.

*Potential issues and solutions*: For some *Bacteroidia* strains that are pretty sensitive to oxygen, the aerobic conjugation will lead to the death of the majority of bacteria, in this case, there are two solutions: (1) expend the *Bacteroidia* strains by recovering the bacteria on the filter in liquid medium (supplemented with antibiotics to suppress the growth of *E. coli*) in the anaerobic chamber, then plate the growth in the liquid medium onto plates with antibiotics to isolate transconjugants; and (2) perform the conjugation on the filter in the anaerobic chamber.

#### Diagnostic PCR and sequencing to verify the single crossover integration

The isolated single colony was inoculated in a 3 mL liquid broth supplemented with 200 µg/mL gentamicin + 15 µg/mL thiamphenicol (or MICs). After 12 h, genomic DNA was extracted using a Quick DNA fungal/bacterial kit (Zymo Research). Diagnostic PCR was performed using primers 16s_27F and R6K_R to verify the single crossover integration of pGM‐NACB/P at their 16s rRNA loci. An ~2.5 kb PCR band would be seen in the transconjugants, whose chromosomal 16s rRNA loci were integrated by pGM‐NACB/P. The 2.5 kb PCR product was purified using a DNA Clean & Concentrator kit (Zymo Research) and sent for sequencing using primer R6K_F_RC. The sequencing results would show that a partial sequence of the 2.5 kb fragment came from the synthetic chi‐16s in pGM‐NACB/P and a partial sequence of the original 16s rRNA gene of the target strain, suggesting a single crossover of pGM‐NACB/P into one of its 16s rRNA loci (Figure 1A).

This single crossover integration strategy readily applies to other genes of interest in *Bacteroidia* microbes. To inactive a target gene in *Bacteroidia* strains, ~1 kb fragment of the target gene is amplified and the purified PCR product is then Gibson‐assembled, with the backbone amplified from the suicide vector pGM‐NACB, to get the plasmid for the target gene (Figure 1A). The plasmid is transferred into the conjugation donor *E. coli* S17 and introduced into the recipient microbe via conjugation. The transconjugants that undergo the expected single crossover integration are identified by diagnostic PCR and sequencing.

Leveraging this protocol, after using the 16s rRNA‐targeting strategy to identify the gene transfer method for a nonmodel gut microbe *Prevotella bivia* DSM 20514, we were able to inactivate the asparaginase gene (*ansB*, which catalyzes the conversion of asparagine to aspartic acid) via single crossover insertion, as shown in Figure 1B,C. In diagnostic PCR, the mutant strain (Δ*ansB*) had the PCR product of ~2 kb, while the wide type (WT) strain had no band (Figure 1B), and we also demonstrated that the mutant strain lost the ability to convert substrate asparagine to aspartic acid by liquid chromatography–mass spectrometry (LC‐MS) (Figure 1C).

On the basis of the essential first step that assesses the tractability of nonmodel *Bacteroidia* microbes, including *Prevotella*, our approach also paves the way for developing more advanced genetic tools, such as the double crossover recyclable marker system. As a proof of concept, after the establishment of the 16s rRNA‐targeting strategy in gut bacteria *Bacteroides* sp. 1_1_30, we further developed the double crossover recyclable marker system to knock out the thymidine kinase gene (*tdk*, which phosphorylates both thymidine and deoxyuridine). As shown in Figure 1D,E, following the integration of the suicide plasmid guided by the left arm and right arm of the targeted gene via HR, the antibiotic marker *catP* will be removed in the second step (double crossover) to get the markerless mutant strain (Figure 1D), in diagnostic PCR, the mutant strain (Δ*tdk*) had the expected shorter PCR product compared with the WT strain (Figure 1E).

*Potential issues and solutions*: If there is no correct transconjugant out of the four restreaked colonies upon diagnostic PCR, suggesting that the integration efficiency is low, in this case, solution 1 is to pick more colonies (like 24 colonies) to restreak and then do diagnostic PCR, solution 2 is to amplify another version of a fragment from the target gene.

*Experimental results interpretation*: For the result of diagnostic PCR of the transconjugants, as shown in Figure 1A,B, because the forward diagnostic primer binds the sequence on the genome and the reverse diagnostic primer binds the sequence on the plasmid, only transconjugants that undergo the expected integration would have the 2.5 kb PCR product; WT strain or transconjugants that undergo the unexpected insertion would not have the 2.5 kb PCR product.

### Identifying methods for *Clostridia* microbes to uptake and stably maintain exogenous genomic DNA

For nonmodel *Clostridia* microbes, culture conditions on agar plates and in liquid broth were screened and identified. For antibiotic resistance, a collection of antibiotics was also screened using a method similar to that used in *Bacteroidia* strains. Likewise, *E. coli* conjugation was used to transport exogenous DNA into the recipient *Clostridia* microbes.

Compared with the *Bacteroidia* strains, the Gram‐positive *Clostridia* gut microbes are more resistant to genetic manipulations for two reasons: (1) It is challenging to deliver exogenous DNA to *Clostridia* strains. *E. coli* conjugation is commonly used to transfer a plasmid with a compatible replication origin (*rep ori*) to a recipient *Clostridia* microbe. The *rep ori* allows the recipient to stably maintain exogenous DNA within the bacteria. (2) *Clostridia* microbes have very inefficient HR.

Previous studies have used the Group II intron (ClosTron) to introduce genome insertion [15] or the CRISPR‐Cas9 to induce chromosomal double‐strand break to promote the selection of HR [10, 11]. Both genetic components need to be assembled with a compatible *rep ori* to be maintained stably in the recipient strain. Therefore, identifying a *Clostridia*‐compatible *rep ori* will be the first step toward developing the genetic tools for *Clostridia* microbes. Their *rep oris* were expanded (from 4 to 9) to identify a compatible *rep ori* for nonmodel gut *Clostridia* strains [22, 23, 24]. A mixed‐conjugation strategy was developed to identify exogenous gene transfer methods for *Clostridia* strains on a large scale.

#### Materials and devices

Primer star DNA polymerase (Takara, Cat# R045), Blue sapphire DNA polymerase (Takara, Cat# RR350), Plasmid Midiprep Kit (Zymo Research, Cat# D4201), DNA Clean and Concentrator (Zymo Research, Cat# D4003), Tryptic Soy Agar (BD, Cat# 236950), Brain Heart Infusion Agar (BD, Cat# 241830), CBA (BD, Cat# 279240), Horse blood (Hemostat Laboratories, Cat# 637291), LB broth (BD, Cat# BP1426), glycerol (Fisher Bioreagents, Cat# BP229), PBS (Gibco, Cat# 10010‐031), centrifuge, PCR amplifier, tetracycline (GoldBio, Cat# T‐101‐25), chloramphenicol (VWR, Cat# 0230), d‐cycloserine (D) (TCI, Cat# C1189), gentamicin (G) (GoldBio, Cat# G‐400‐25), kanamycin (K) (GoldBio, Cat# K‐120‐25), carbenicillin (GoldBio, Cat# C‐103‐25), thiamphenicol (Thiam) (Acros Organics, Cat# 455450250), anaerobic chamber, aerobic incubator, electroporation system, Thermo Scientific Nanodrop 2000, Gibson Assembly Cloning Kit (NEB, Cat# E5510S), Quick DNA fungal/bacterial kit (Zymo Research, Cat# D6005), and ultralow temperature freezer.

#### Screening of culture conditions

**Figure 2:**
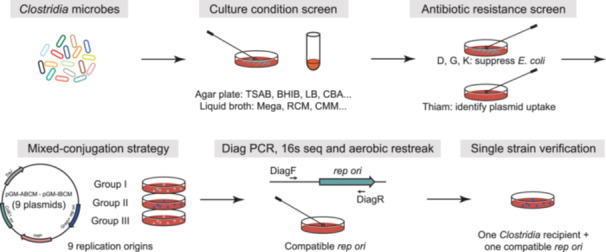
Workflow for the identification of methods for *Clostridia* microbes to uptake and stably maintain exogenous genomic DNA. BHIB, Brain Heart Infusion Agar + Horse blood; CBA, Columbia Blood Agar; CMM, Chopped Meat Medium; D, d‐cycloserine; *E. coli*, *Escherichia coli*; G, gentamicin; K, kanamycin; LB, Luria–Bertani; PCR, polymerase chain reaction; RCM, Reinforced Clostridial Medium; *rep ori*, replication origin; TSAB, Tryptic Soy Agar + Horse blood.

The culture conditions for the Gram‐positive *Clostridia* microbes were screened. Strains were restreaked (from the original glycerol stock) onto pre‐reduced agar plates (such as TSAB, BHIB, or CBA plates). Then, microbes that can grow on these agar plates were subcultured into 1 mL pre‐reduced liquid medium: Mega, CMM, and RCM. Strains that can grow in any one of the four liquid cultures were subject to the antibiotics test (Figure 2).

*Keynotes*: For the screening of the culture conditions of liquid medium, *Clostridia* strains need to be first recovered on agar plates to ensure that *Clostridia* strains are activated, instead of inoculating *Clostridia* strains into the liquid medium from the frozen glycerol stock directly.

*Potential issues and solutions*: If the *Clostridia* strains of interest cannot grow on the common agar plates or in the liquid medium listed above, other specific plates or liquid mediums that favor the growth of the target strains need to be used to screen the culture conditions.

#### Antibiotic test

The *Clostridia* strains were restreaked on agar plates supplemented with 250 µg/mL d‐cycloserine (D), 200 µg/mL gentamicin (G), or 200 µg/mL kanamycin (K). d‐cycloserine or gentamicin will be used to inhibit the growth of conjugation donor *E. coli* CA434 after conjugation, and kanamycin will be used to inhibit the growth of conjugation donor *E. coli* HB101/pRK24. Both *E. coli* CA434 and HB101/pRK24 have been shown to successfully conjugate exogenous genomic DNA into *Clostridium* bacteria like *Clostridium sporogenes* or *Clostridium acetobutylicum* in previous studies [10, 11, 15].

The majority of the *Clostridia* strains we tested so far are sensitive to thiamphenicol, so the thiamphenicol‐resistant gene (*catP*) can be exploited as a universal marker to select transconjugants that can uptake and maintain exogenous genomic DNA. The MICs of thiamphenicol of the *Clostridia* microbes were tested on agar plates containing thiamphenicol at different concentrations (Figure 2).

*Keynotes*: Antibiotics need to be added into the agar medium before the agar plates are poured and solidified; agar plates supplemented with antibiotics only on the surface of the plates may cause misleading antibiotic test results.

*Potential issues and solutions*: If the *Clostridia* strains of interest are not resistant against d‐cycloserine, gentamicin, or kanamycin, other specific antibiotics that can suppress the growth of *E. coli* CA434 or HB101/pRK24 can be used for the test. Likewise, if the *Clostridia* strains of interest are resistant against thiamphenicol, the antibiotic marker on the conjugation vector can be replaced by other markers, whose corresponding antibiotics can suppress the growth of the *Clostridia* strains.

#### Vector assembly

A series of vectors pGM‐xBCM (pGM‐ABCM, BBCM, CBCM, DBCM, EBCM, FBCM, GBCM, HBCM, and IBCM) harboring different *rep oris* for *Clostridia* microbes were generated to screen the compatible *rep oris* for the recipient *Clostridia* strains [2]. To include more *rep oris* for screening, the *rep ori* sequences could be amplified by PCR and assembled with the backbone amplified from pGM‐xBCM.

#### Mixed‐conjugation strategy to identify *Clostridia* microbes that uptake and maintain exogenous plasmid DNA

The series of vectors pGM‐ABCM, BBCM, CBCM, DBCM, EBCM, FBCM, GBCM, HBCM, and IBCM harboring different *rep oris*, but the same antibiotic marker *catP* (against thiamphenicol) were transformed into chemical competent *E. coli* CA434 or *E. coli* HB101/pRK24. Mixed‐conjugation strategies separating these *E. coli* donors into three groups (Group I: pGM‐ABCM, BBCM, and CBCM; Group II: pGM‐DBCM, EBCM, and FBCM; and Group III: pGM‐GBCM, HBCM, IBCM) were established to identify the compatible *rep ori* for each *Clostridia* microbe of interest. For the *Clostridia* microbes resistant to d‐cycloserine (250 μg/mL) or gentamicin (200 µg/mL), *E. coli* CA434 would be used as the conjugation donor. For the microbes that are not resistant to d‐cycloserine (250 μg/mL) or gentamicin (200 µg/mL) but resistant to kanamycin (200 μg/mL), *E. coli* HB101/pRK24 would be used as their conjugation donors.

The *Clostridia* microbe was restreaked on a pre‐reduced agar plate. After 24–48 h, a single colony was inoculated in 1 mL of liquid broth that supported its growth in an anaerobic chamber. On the same day, *E. coli* containing plasmids with different *rep oris* were inoculated into 6 mL of LB supplemented with tetracycline (15 µg/mL) and chloramphenicol (25 µg/mL) and shaken aerobically at 37°C for 12–18 h (overnight). The next day, these *E. coli* donors were separated into three groups, as mentioned above. For conjugating one *Clostridia* microbe, a 1.0 mL culture of each *E. coli* within the same group was mixed and centrifuged at 1500*g* for 2 min. The culture supernatant was discarded, and the cell pellet was gently washed with 500 µL PBS buffer (pH = 7.4). The PBS supernatant was then removed after centrifugation at 1500*g* for 2 min, and the cell pellet was transferred on ice into the anaerobic chamber. Next, the cell pellet (a total of three cell pellets) was mixed gently with 300 μL overnight culture of the targeting *Clostridia* microbe, and a 35 μL cell mixture was dotted on pre‐reduced agar plates. After 48 h, the cell dots were scraped using a sterile inoculation loop and resuspended in 300 μL pre‐reduced PBS (pH = 7.4) buffer. The cell suspension (100 µL) was plated on agar plates supplemented with 15 µg/mL thiamphenicol (or MICs) and 250 µg/mL d‐cycloserine or 200 µg/mL gentamicin (if *E. coli* CA434 is the conjugation donor), or 200 µg/mL kanamycin (if *E. coli* HB101/pRK24 is the conjugation donor). Colonies typically appeared after 36–48 h. Four colonies were picked and restreaked onto agar plates with the same antibiotics to isolate single colonies (Figure 2).

*Keynotes*: (1) The target *Clostridia* microbes are cultured in the anaerobic chamber overnight (~12 h), do not culture the *Clostridia* strains for too long before conjugation, which will lead to the lysis of the strains; (2) air dry the agar plates for conjugation a little bit is good for conjugation, if the plates are wet, the cell mixture dot will spread all over the plate, in this case, the cell mixture is diluted on the plate, which will reduce the conjugation efficiency.

*Potential issues and solutions*: In our experience, conjugation donor *E. coli* CA434 works better than *E. coli* HB101/pRK24. For specific strains of interest, if the two *E. coli* donors cannot transfer plasmids into the recipient strains, other *E. coli* donors could be utilized.

#### Diagnostic PCR and sequencing to verify the plasmid uptake

The isolated single colony was cultivated in 3 mL liquid broth supplemented with the corresponding antibiotics 250 µg/mL d‐cycloserine (or 200 µg/mL gentamicin/kanamycin) + 15 µg/mL thiamphenicol (or MICs). The genomic DNA was isolated from the resulting cell material using the Quick DNA fungal/bacterial kit (Zymo Research). Then multiplex diagnostic PCRs were performed to assess which plasmid was uptaken by the conjugation recipient *Clostridia* microbe. For the mixed‐conjugation with Group I (Groups II and III are performed likewise), primers pMTL_*laz*_diag_F (universal forward primer) + pGM‐ABCM_rep_R_1500bp + pGM‐BBCM_rep_R_1000bp + pGM‐CBCM_rep_R_2000bp (for 15 µL PCR reaction, the amount of the four primers is 0.75, 0.3, 0.3, and 0.3 µL [10 µM]) were used for diagnostic PCR. A PCR band of 1.5 kb (or 1.0 or 2.0 kb) would be seen if pGM‐ABCM (or BBCM or CBCM) is uptaken by the *Clostridia* microbe. In the meantime, to confirm that the picked and restreaked colonies are the target *Clostridia* strain but not the *E. coli* conjugation donor, the 16s rRNA region of the colony was amplified using primers 16s_27F + 16s_1391R. The PCR product was purified and sent for Sanger sequencing using primer 16s_1391R, and the colonies were further restreaked aerobically to confirm not to be *E. coli* (if the colonies cannot grow aerobically, they will be considered not to be *E. coli*) (Figure 2).

*Keynotes*: When performing the diagnostic PCR to figure out which plasmid in a group was transferred into the recipient strain, to avoid a false‐positive conclusion, it is necessary to include the genome of the recipient strain to serve as the negative control.

*Experimental results interpretation*: For the diagnostic PCR of each group (I, II, and III), when different plasmids are transferred into recipient strains, there will be PCR products of different sizes (1, 1.5, and 2 kb), the corresponding size of each plasmid in each group is annotated at the end of the “Name of primers” in Table S1.

#### Single *E. coli* donor‐conjugation validation

Next, single *E. coli* donor‐conjugation (one *E. coli* donor to one *Clostridia* recipient) was performed to validate that the PCR‐identified plasmid(s) can be conjugated into the targeted *Clostridia* microbe. A single colony of the targeted *Clostridia* strain was inoculated in a 1 mL liquid broth in an anaerobic chamber. The conjugation donor *E. coli* (CA434 or HB101/pRK24) harboring the PCR‐identified plasmid was inoculated into 6 mL of LB supplemented with tetracycline (15 µg/mL) and chloramphenicol (25 µg/mL) and shaken aerobically at 37°C for 12–18 h (overnight). After 12–18 h, 1.5 mL of the *E. coli* culture was centrifuged at 1500*g* for 2 min. The supernatant was discarded and the cell pellet was washed with 500 µL PBS buffer (pH = 7.4). The PBS supernatant was then removed after centrifugation at 1500*g* for 2 min, and the cell pellet was transferred on ice into the anaerobic chamber. Next, the cell pellet was mixed gently with a 300 μL overnight culture of the targeting *Clostridia* microbe, and a 35 μL cell mixture was dotted on pre‐reduced agar plates. After 48 h, the cell dots were scraped using a sterile inoculation loop and resuspended in 300 μL pre‐reduced PBS (pH = 7.4) buffer. The cell suspension (100 µL) was plated on agar plates supplemented with 15 µg/mL thiamphenicol (or MICs) and 250 µg/mL d‐cycloserine or 200 µg/mL gentamicin (if *E. coli* CA434 is the conjugation donor), or 200 µg/mL kanamycin (if *E. coli* HB101/pRK24 is the conjugation donor). Colonies typically appeared after 36–48 h. Four colonies were picked and restreaked onto agar plates with the same antibiotics to isolate single colonies. The isolated single colonies will be cultured in 1 mL of pre‐reduced liquid broth with the same antibiotics, and the glycerol stock will be prepared using the culture (Figure 2).

*Keynotes*: For the step of scraping cell dots and suspending cells in PBS to be plated onto agar plates with selective antibiotics, in the case that a lot of conjugations plates need to be scraped, it is better to scrap at most three plates and suspend in PBS at one time, keeping cells in PBS for too long will reduce the conjugation efficiency.

*Potential issues and solutions*: In the step of plating conjugation cells onto agar plates with d‐cycloserine (or gentamicin, kanamycin) + thiamphenicol (MICs), some recipient strains may overgrow because the MICs of thiamphenicol are not enough to suppress the growth of the plated cells that contain a high concentration of recipient strains; in this case, plates with a higher concentration of thiamphenicol need to be used (e.g., if the MICs of thiamphenicol are 7.5 µg/mL, 10 µg/mL thiamphenicol can be added into the agar plate).

### Developing a CRISPRi‐dCpf1 *lacZ*α system for *Clostridia* microbes

After identifying exogenous plasmid transfer methods for *Clostridia* strains, the next step is developing a tractable genetic tool that would function in those *Clostridia* commensals. Like Cas9‐mediated cutting and dCas9‐induced interference, CRISPR‐based genome editing systems have been recently used to manipulate *C. sporogenes* [10, 11] and *Clostridium difficile* [25]. In general, *Clostridia* has very inefficient HR, and the DNA double‐stranded break initiated by Cas9 or the like is mostly lethal. While much effort was spent finetuning a spectrum of conjugation parameters to identify the optimal condition for the Cas9 machinery in *C. sporogenes*, this condition is usually not readily applicable to other *Clostridia* commensals.

CRISPR interference deactivated Cpf1 (CRISPRi‐dCpf1) [26, 27, 28, 29, 30, 31] system was prioritized for *Clostridia* microbes because the dCpf1 does not initiate the DNA double‐strand break and is supposedly less toxic and applicable to a broader range of *Clostridia* compared with the Cas9/Cpf1. Indeed, plasmids carrying dCpf1 showed less toxicity and relatively higher conjugation efficiency than those with Cas9 or Cpf1. Combined with the CRISPRi‐dCpf1 machinery, *lacZα* was used as a transcription reporter to develop CRISPRi‐dCpf1 gene repression tools for nonmodel *Clostridia* microbes without prior knowledge of the genome sequence.

#### Materials and devices

Primer star DNA polymerase (Takara, Cat# R045), Blue sapphire DNA polymerase (Takara, Cat# RR350), Plasmid Midiprep Kit (Zymo Research, Cat# D4201), DNA Clean and Concentrator (Zymo Research, Cat# D4003), Tryptic Soy Agar (BD, Cat# 236950), Brain Heart Infusion Agar (BD, Cat# 241830), CBA (BD, Cat# 279240), Horse blood (Hemostat Laboratories, Cat# 637291), LB broth (BD, Cat# BP1426), glycerol (Fisher Bioreagents, Cat# BP229), PBS (Gibco, Cat# 10010‐031), centrifuge, PCR amplifier, tetracycline (GoldBio, Cat# T‐101‐25), chloramphenicol (VWR, Cat# 0230), d‐cycloserine (D) (TCI, Cat# C1189), gentamicin (G) (GoldBio, Cat# G‐400‐25), kanamycin (K) (GoldBio, Cat# K‐120‐25), thiamphenicol (Thiam) (Acros Organics, Cat# 455450250), anaerobic chamber, aerobic incubator, electroporation system, Thermo Scientific Nanodrop 2000, Gibson Assembly Cloning Kit (NEB, Cat# E5510S), Quick DNA fungal/bacterial kit (Zymo Research, Cat# D6005), ultralow temperature freezer, Direct‐zol RNA Microprep kit (Zymo Research, Cat# R2062), PrimeScript RT Reagent Kit (Takara, Cat# RR047A), real‐time quantitative PCR system (Applied Biosystems, ABI 7500).

#### Vector assembly

**Figure 3:**
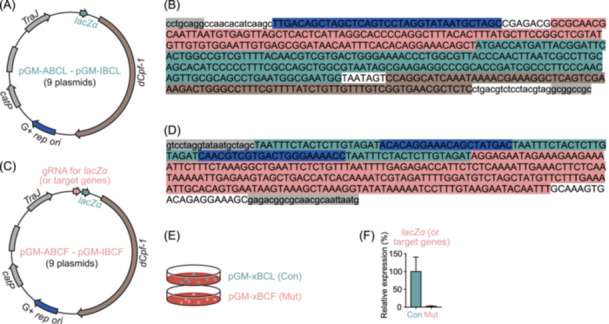
Development of a CRISPRi‐dCpf1 *lacZα* genetic manipulation system for *Clostridia* microbes. (A) Schematics of the set of plasmids pGM‐ABCL–pGM‐IBCL that carry the clustered regularly interspaced short palindromic repeats interference deactivated Cpf1 (CRISPRi‐dCpf1) machinery and the *lacZα* reporter gene. G+ *rep ori*, Gram‐positive replication origin. (B) The sequence of the *lacZα* locus consisting of guide RNA (gRNA) promoter P_J23119_ (highlighted in blue), the *lacZα* promoter (in red), the *lacZα* coding sequence (in green), and *lacZα* terminator (in brown). The sequences highlighted in gray are restriction sites of SbfI and NotI, respectively. (C) Schematics of the set of plasmids pGM‐ABCF–pGM‐IBCF that carry the CRISPRi‐dCpf1 machinery, the *lacZα* reporter gene, and gRNA locus targeting the promoter region and coding sequence (CDS) of *lacZα*. (D) The sequence of the gRNA locus consisting of three dCpf1 direct repeat sequences (highlighted in green), two gRNA targeting both the promoter region and the template strand of *lacZα* (in blue), and terminator region obtained from the 16s rRNA gene of *Clostridium sporogenes* ATCC 15579 (*CLOSPO_00916*) (in red). The sequences highlighted in gray are homologous to the sequence in pGM‐ABCL. (E, F) Plasmids with compatible replication origins in the set of plasmids pGM‐ABCL–pGM‐IBCL (pGM‐xBCL, Control group, Con) and pGM‐ABCF–pGM‐IBCF (pGM‐xBCF, Mutant group, Mut) were introduced into the recipient *Clostridia* microbes, and the expression levels of *lacZα* were quantified by quantitative polymerase chain reaction (qPCR).

Three sets of plasmids pGM‐xBCD (pGM‐ABCD, BBCD, pGM‐CBCD, DBCD, EBCD, FBCD, GBCD, HBCD, and IBCD) carrying the CRISPRi‐dCpf1 machinery, plasmids pGM‐xBCL (pGM‐ABCL, BBCL, pGM‐CBCL, DBCL, EBCL, FBCL, GBCL, HBCL, and IBCL) carrying CRISPRi‐dCpf1 and the *lacZα* reporter gene, and plasmids pGM‐xBCF (pGM‐ABCF, BBCF, pGM‐CBCF, DBCF, EBCF, FBCF, GBCF, HBCF, and IBCF) carrying CRISPRi‐dCpf1, *lacZα*, and the *lacZα* targeting guide RNA (gRNA) were generated to test the CRISPRi‐dCpf1 *lacZα* system for *Clostridia* microbes [2] (Figure 3A–D). To target other specific genes in *Clostridia* strains, the gRNA locus targeting the promoter region and coding sequence (CDS) of the target gene (as in Figure 3D) could be amplified by PCR and assembled with the backbone amplified from pGM‐xBCD to get the plasmid carrying CRISPRi‐dCpf1 and the gRNA for the target gene.

#### Utilization of dCpf1 to suppress the *lacZα* transcription in *Clostridia* strains

Using a Gram‐positive strain *Clostridium bolteae* DSM 29485 as an example, pGM‐ABCL and pGM‐ABCF were transformed into chemically competent *E. coli* CA434, respectively. *E. coli* CA434 harboring pGM‐ABCL and pGM‐ABCF were conjugated to *Clostridium bolteae* DSM 29485 (Figure 3E). The transconjugants were picked and restreaked onto a TSAB agar plate supplemented with d‐cycloserine (250 µg/mL) + thiamphenicol (15 µg/mL). Then, three isolated single colonies were cultivated in 5 mL Mega liquid broth supplemented with 15 µg/mL thiamphenicol for 36 h. The bacterial RNA was extracted using a Direct‐zol RNA Microprep kit (Zymo Research) and quantitative polymerase chain reaction (qPCR) was performed to quantify the relative expression of *lacZα* after normalizing to 16s rRNA gene, using primers dCpf1‐*lacZα*_qPCR_F and dCpf1‐*lacZα*_qPCR_R for *lacZα* gene and S74_16 s_qPCR_F and S74_16s_qPCR_R for the control 16s rRNA (Figure 3F).

This CRISPRi‐dCpf1 gene repression tool is readily applicable to other genes of interest in *Clostridia* microbes. To knock down a target gene in *Clostridia* strains, the gRNA locus targeting the promoter region and CDS of the target gene is introduced into the plasmid harboring dCpf‐1 and the corresponding compatible *rep ori* (the set of vectors pGM‐xBCD). As in targeting *lacZα*, the synthetic fragment containing the terminator region (Figure 3D, in red) was amplified to get a PCR product that has one direct repeat sequence (Figure 3D, in green) and gRNA (Figure 3D, in blue) fused with the terminator. Then, this PCR product was purified and used as the template for the second PCR to get the gRNA locus with two gRNAs for the target gene. This gRNA locus was then assembled with the backbone amplified from pGM‐xBCD to get the plasmid for the target gene. The plasmid was transformed into donor *E. coli* CA434 (or HB101/pRK24) and introduced into the recipient microbe via conjugation. The transconjugants harboring the CRISPRi‐dCpf1 plasmid are identified by antibiotic selection, and the gene knockdown is validated by qPCR of the target gene or other readout like LC‐MS measurement of metabolites.

*Keynotes*: To transfer plasmids containing the CRISPRi‐dCpf1 machinery into the target strains, in the step of plating conjugation cells onto agar plates with selective antibiotics, it is recommended to plate all 300 µL scraped‐cell suspension in PBS onto three plates, because the conjugation efficiency will decrease when the CRISPRi‐dCpf1 machinery is introduced into plasmids to make these plasmids bigger.

*Potential issues and solutions*: In some cases, the knockdown efficiency is low because the CRISPRi‐dCpf1 system has off‐target effects. To address this problem, one option is to test several different gRNA designs (construct different plasmids containing different gRNAs); the other option is to construct multiple gRNAs containing plasmids, an extended version of our current duplex gRNA design, introducing multiple gRNAs, like four gRNAs, would reduce the off‐target effects and enhance the suppressive efficiency of the CRISPRi‐dCpf1 system.

*Experimental results interpretation*: To determine the suppressive efficiency of target genes by the CRISPRi‐dCpf1 system, the expression of target genes is quantified by qPCR and normalized to reference 16s rRNA gene of the strain. In the case the introduced *lacZα* gene is the target gene, the strain harboring plasmid pGM‐xBCL (carrying CRISPRi‐dCpf1 + *lacZα*) serves as the control, and expression of the *lacZα* gene in the strain harboring plasmid pGM‐xBCF (carrying CRISPRi‐dCpf1 + *lacZα* + *lacZα* targeting gRNAs) is normalized to the expression of *lacZα* gene in control (as shown in Figure 3F). In the case, where a specific gene on the genome of the strain of interest is the target gene, the strain harboring plasmid pGM‐xBCD (carrying CRISPRi‐dCpf1) serves as the control, and expression of the target gene in the strain harboring plasmids that carry CRISPRi‐dCpf1 + gene targeting gRNAs is normalized to the expression of the target gene in control.

### Developing a 16s‐tron strategy for *Clostridia* microbes

Group II intron‐based genetic tools were also developed for nonmodel gut *Clostridia* microbes. Group II intron was selected because it facilitates the insertion of retrotransposition‐activated markers (RAMs) into the targeted genome sites [15]. This targeted insertion does not induce lethal chromosomal double‐strand breaks (as initiated by Cas9) that need to be repaired by HR.

As in *Bacteroidia* microbes, conserved bacterial 16s rRNA genes of *Clostridia* strains were selected as a target gene to develop genetic manipulation tools without prior knowledge of their genome sequences. Multiple sequence alignment using 16s rRNAs of *Clostridia* that can uptake plasmids was performed to identify a highly conserved sequence that can be targeted by Group II intron. Then, the intron targeting and design tool on the ClosTron website (https://clostron.com/intron-design-tool) was used to design the Group II introns targeting the conserved 16s sequence. For each recipient *Clostridia* microbe, the designed 16s‐targeting Group II intron (16s‐tron) was assembled with their compatible *rep oris* and antibiotic RAM and transported into the recipient *Clostridia* microbes; the RAM provides antibiotic resistance only upon integration into the *Clostridia* chromosome.

#### Materials and devices

Primer star DNA polymerase (Takara, Cat# R045), Blue sapphire DNA polymerase (Takara, Cat# RR350), Plasmid Midiprep Kit (Zymo Research, Cat# D4201), DNA Clean and Concentrator (Zymo Research, Cat# D4003), Tryptic Soy Agar (BD, Cat# 236950), Brain Heart Infusion Agar (BD, Cat# 241830), CBA (BD, Cat# 279240), Horse blood (Hemostat Laboratories, Cat# 637291), LB broth (BD, Cat# BP1426), glycerol (Fisher Bioreagents, Cat# BP229), PBS (Gibco, Cat# 10010‐031), centrifuge, PCR amplifier, tetracycline (GoldBio, Cat# T‐101‐25), chloramphenicol (VWR, Cat# 0230), d‐cycloserine (D) (TCI, Cat# C1189), gentamicin (G) (GoldBio, Cat# G‐400‐25), kanamycin (K) (GoldBio, Cat# K‐120‐25), thiamphenicol (Thiam) (Acros Organics, Cat# 455450250), erythromycin (VWR, Cat# 0219), spectinomycin (Cat# BML‐A281‐0010), anaerobic chamber, aerobic incubator, electroporation system, Thermo Scientific Nanodrop 2000, Gibson Assembly Cloning Kit (NEB, Cat# E5510S), Quick DNA fungal/bacterial kit (Zymo Research, Cat# D6005), and ultralow temperature freezer.

#### Vector assembly

**Figure 4:**
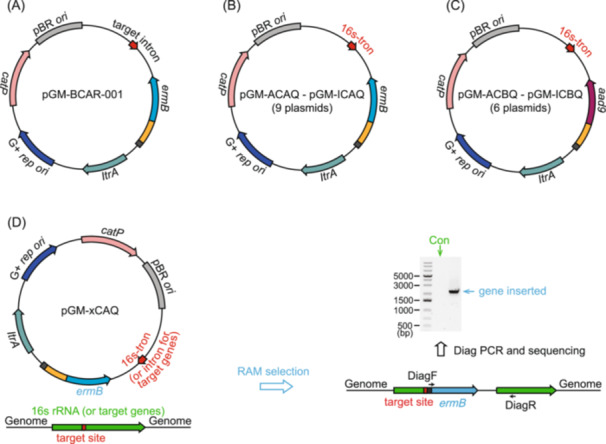
Development of a 16s‐tron genetic manipulation strategy for *Clostridia* microbes. (A) Schematics of the starting Group II intron plasmid pGM‐BCAR‐001, which was previously assembled for the insertion of one target gene in the genome of *Clostridium sporogenes* ATCC 15579 (shown as “target intron”), with *catP* as the conjugation‐selection marker and *ermB* as the retrotransposition‐activated marker (RAM). G+ *rep ori*, Gram‐positive replication origin. (B, C) Schematics of the set of plasmids pGM‐ACAQ–pGM‐ICAQ and pGM‐ACBQ–pGM‐ICBQ carrying the 16s‐targeting Group II intron (16s‐tron), which mediate integration of the RAM into the targeted 16s rRNA genes, with *catP* as the conjugation‐selection marker, *ermB* (B) and *aad9* (C) as the RAM. (D) Diagnostic PCR strategy to validate the 16s‐tron RAM integration into the 16s rRNA genes of targeted *Clostridia* microbes. The DiagF is the sequence on the RAM, which will not bind to the genome. The DiagR binds to the genome and will not bind to the Group II intron plasmid. There will be a PCR product of 2.0–2.5 kb as designed for colonies that have integrated the RAM, whereas no PCR product will be found for control colonies. PCR, polymerase chain reaction; rRNA, ribosomal RNA.

Two sets of plasmids (1) pGM‐xCAQ (pGM‐ACAQ, BCAQ, CCAQ, DCAQ, ECAQ, FCAQ, GCAQ, HCAQ, and ICAQ) whose conjugation‐selection marker is *catP*, and RAM is *ermB*, and (2) plasmids pGM‐xCBQ (pGM‐ACBQ, DCBQ, ECBQ, FCBQ, HCBQ, and ICBQ) whose conjugation‐selection marker is *catP*, and RAM is *aad9*, were generated to test the 16s‐tron strategy for *Clostridia* microbes [2] (Figure 4A–C). To target other specific genes in *Clostridia* strains, the introns for the target gene (~300 bp, Figure 4D) could be designed using the ClosTron website and synthesized and assembled with the backbone amplified from pGM‐xCAQ to get the plasmid for the target gene.

*Keynotes*: Different combinations of conjugation‐selection markers and RAM could be selected and used to replace the antibiotic markers in those established plasmids.

#### Introduction of the assembled 16s‐tron vectors and selection of the RAM‐integrated mutants

Using the strain *Blautia luti* DSM 14534 (S54) as an example, the plasmid pGM‐FCAQ was transformed into chemically competent *E. coli* CA434. Then *E. coli* CA434 harboring plasmid pGM‐FCAQ was conjugated to S54. The transconjugants were picked and restreaked onto a TSAB agar plate supplemented with d‐cycloserine (250 µg/mL) + thiamphenicol (15 µg/mL). Then, three single colonies were cultivated into 1 mL Mega liquid broth supplied with 15 µg/mL thiamphenicol and 250 µg/mL d‐cycloserine. After 24–36 h, 50 µL of cultures were spread onto TSAB agar plates supplemented with 250 µg/mL d‐cycloserine and 10 µg/mL erythromycin. The transconjugants typically appeared after 36–48 h. Eight colonies were picked to inoculate 3 mL Mega liquid broth supplemented with 250 µg/mL d‐cycloserine and 10 µg/mL erythromycin. After 24–36 h, genomic DNA was extracted using Quick DNA fungal/bacterial kit (Zymo Research) and diagnostic PCR was performed using primers 16s_tron_diagR_v4 + 16s_1391R + 16s_1391R_3to5 (with 16s_tron_diagR_v4 binding the integrated intron part and 16s_1391R + 16s_1391R_3to5 binding the target 16s site, only colonies that undergo RAM integration will have the band of ~2.5 kb) (Figure 4D).

This Group II intron strategy also readily applies to other genes of interest in *Clostridia* microbes. To mutate a target gene in *Clostridia* strains, the introns for the target gene (~300 bp, Figure 4D) are designed using the design tool on the ClosTron website and synthesized. The synthesized fragment was assembled with the backbone amplified from the set of plasmids pGM‐xCAQ with a compatible *rep ori* and antibiotic marker to get the plasmid for mutating the target gene. The plasmid was transferred into donor *E. coli* CA434 (or HB101/pRK24) and introduced into the recipient microbe via conjugation. The transconjugants harboring the plasmid are identified by antibiotic selection with d‐cycloserine and thiamphenicol. Then, colonies are cultivated into liquid broth with d‐cycloserine and thiamphenicol, and 50 µL of cultures are spread onto agar plates supplemented with d‐cycloserine and erythromycin (or other antibiotic markers to select for RAM insertion). The insertion mutants are validated by diagnostic PCR and sequencing as with 16s rRNA.

*Keynotes*: The Group II intron‐based plasmids are about 10 kb, which is much bigger than the plasmids used for the screening of plasmid uptake and will reduce the conjugation efficiency, in the step of plating conjugation cells onto agar plates with selective antibiotics, it is better to plate all 300 µL scraped‐cell suspension in PBS onto three different plates (100 µL onto each plate).

*Potential issues and solutions*: For some strains, the efficiency of the RAM selection step is low, and the above‐mentioned plating volume (50 µL) is not enough, in this case, more liquid could be plated onto the RAM selection plates, and is recommended to plate several plates.

*Experimental results interpretation*: For the result of diagnostic PCR of the transconjugants up on RAM selection, as shown in Figure 4D, because the forward diagnostic primer binds the sequence on the plasmid and the reverse diagnostic primer binds the sequence on the genome, only transconjugants that undergo the expected integration would have the 2.5 kb PCR product; WT strain or transconjugants that undergo the unexpected insertion would not have the 2.5 kb PCR product.

## METHODS

Configuration methods and formulas for key solution reagents and medium is available in the Supporting Information.

## SUMMARY

Many gut microbiota genes are associated with diseases like inflammatory bowel disease and colon cancer [1, 2, 3, 4, 5, 6], yet it is challenging to causally dissect their contributions at the molecular level because most gut commensals are nonmodel and genetically intractable. The genetic manipulation methods we introduce here provide an efficient and potentially generalizable microbiota genetic tool screening pipeline to screen nonmodel gut commensals and establish their tractable genetic systems on a large scale.

The bacterial 16s rRNA genes have long been used to reconstruct phylogenies and assess microbiome diversity. Since the *Bacteroidia* has relatively higher efficiency in HR, their highly conserved 16s rRNA gene could instead serve as an “archery target” to be inserted by the introduced suicide vector through a single crossover. Likewise, the 16s rRNA genes could also serve as a universal target in *Clostridia* microbes to be integrated by the 16s‐targeting Group II intron (16s‐tron) plasmid containing compatible *rep oris* and antibiotic RAM. Furthermore, CRISPR machinery targeting *lacZα* transcription in the introduced plasmid (CRISPRi‐*lacZα* system) could also be applied to establish the genetic tools in nonmodel *Clostridia* strains.

The pipeline we provide has three notable features. First, by targeting the 16s rRNA gene or assembling a CRISPRi‐dCpf1 *lacZα* system, the genetic systems could be built in gut bacteria without prior knowledge of their genome information. Second, without the “tune and test” process, the pipeline builds tractable genetic toolsets for multiple nonmodel *Bacteroidia* and *Clostridia* within weeks. Third, the pGM vectors are modular, and different genetic components, such as chimeric‐16s, *rep oris*, tagging markers like green fluorescent protein, or other nonnative genes of interesting biological function, can be switched/combined and introduced into these nonmodel gut commensals. All three features suggest the potential of the pipeline as a high‐throughput genetics screening and manipulating platform for the human gut microbiome.

Despite these advanced features, our strategies have limitations. First, the *Bacteroidia* genes are mutated via single crossover integration which sometimes leads to only partial dysfunction of their proteins, and this single crossover integration strategy would not work well when the size of the target gene is too small. In these cases, double crossover design could serve as a backup plan, with the left and right flanks of the target gene used for the single crossover, and double crossover comes later to get the expected knockout, our single crossover integration screening protocol can help pave the way for developing marker recycling system via double crossover in *Bacteroides*. Second, the CRISPRi‐dCpf1 system has off‐target effects; several gRNAs in the dCpf1 system might need to be tested to efficiently repress the target gene. Multiple gRNA design, an extended version of our current duplex gRNA design, may help overcome this limitation. Introducing multiple gRNAs, like four gRNAs, would reduce the off‐target effects and enhance the suppressive efficiency of the CRISPRi‐dCpf1 system [32, 33].

Although all the strategies described above are developed to screen and establish genetic manipulation systems in *Bacteroidia* and *Clostridia* strains on a relatively large scale, our protocol is applicable to microbes from other Phyla except *Bacteroidia* and *Clostridia*. For example, the single crossover integration strategy for *Bacteroidia* microbes using suicide plasmid works well in other Gram‐negative gut microbes. We have proved that suicide plasmid targeting strain‐specific 16s rRNA genes can be integrated into the targeted site of the genome of other Gram‐negative gut microbes, such as *Fusobacterium gastrosuis* DSM 101753, *Fusobacterium nucleatum* ATCC 25751, ATCC 10953, ATCC 23726, *Klebsiella oxytoca* DSM 29614, DSM 5175, DSM 7342, *Proteus mirabilis* ATCC 35659, and *Proteus vulgaris* DSM 3265. Also, the strategy of screening compatible *rep ori*‐harboring plasmids works in other Gram‐positive gut microbes. In Gram‐positive gut microbes like *Bifidobacterium catenulatum* DSM 16992 and several *Enterococcus faecalis* strains, we screened the compatible *rep ori*‐harboring plasmids and showed that the CRISPRi‐dCpf1 *lacZα* system worked well in those strains [34]. Furthermore, these strategies are readily applicable to other genes of interest (instead of 16s rRNA or *lacZα*, shown as “target genes” in Figures 1, 3, and 4) in other microbes, and the feasibility and reliability of the genetic manipulation strategies described here have been verified by multiple studies [34, 35]. This high‐throughput and generalizable protocol will greatly facilitate the molecular mechanism investigation of gut microbiota‐host interaction from the following aspects: (1) development of genetic tools in nonmodel bacteria that may be physiologically important, (2) precise manipulation of microbiota genes to assess their effect on host metabolism and biology, (3) disclosure of biosynthesis of microbiota‐derived metabolites like deoxycholic acid, and (4) stimulation of new strategies to engineer microbiota at the single gene level.

## AUTHOR CONTRIBUTIONS

Chun‐Jun Guo and Wen‐Bing Jin conceived the protocol and designed the experiments. Wen‐Bing Jin performed the experiments and analyzed the data. Wen‐Bing Jin wrote the manuscript. Chun‐Jun Guo revised the manuscript and supervised this project. All authors have read the final manuscript and approved it for publication.

## CONFLICT OF INTEREST STATEMENT

The authors declare no conflict of interest.

## Supporting information


**Table S1.** Primers used in this protocol.

Supporting information.

## Data Availability

Data sharing is not applicable to this article as no new data were created or analyzed in this study. Supplementary materials (figures, tables, graphical abstract, slides, videos, Chinese translated version, and update materials) may be found in the online DOI or iMeta Science http://www.imeta.science/.
