# Supplementary material for: Genetic manipulations of nonmodel gut microbes
Source: Imeta. 2024 Jun 23;3(4):e216. doi: 10.1002/imt2.216 (PMC11316930; doi:10.1002/imt2.216)
Supplement: Supplementary file 2 — Supporting information. [file IMT2-3-e216-s002.docx]

**Supporting information to**

**Genetic manipulations of** **nonmodel gut microbes**

**Running title**: Genetic tools for nonmodel gut microbes

Wen-Bing Jin^1,2^* and Chun-Jun Guo^1,2,3,4,5^*

^1^Jill Roberts Institute for Research in Inflammatory Bowel Disease, Weill Cornell Medicine, Cornell University, New York, NY 10021, USA

^2^Friedman Center for Nutrition and Inflammation, Weill Cornell Medicine, Cornell University, New York, NY 10021, USA

^3^Joan and Sanford I. Weill Department of Medicine, Gastroenterology and Hepatology Division, Weill Cornell Medicine, Cornell University, New York, NY 10021, USA

^4^Department of Microbiology and Immunology, Weill Cornell Medicine, Cornell University, New York, NY 10021, USA

^5^Immunology and Microbial Pathogenesis Program, Weill Cornell Graduate School of Medical Sciences, Weill Cornell Medicine, Cornell University, New York, NY 10065, USA

*Correspondence: [wej4002@med.cornell.edu](mailto:wej4002@med.cornell.edu) (Wen-Bing Jin) and [cj@guo-group.org](mailto:cj@guo-group.org) (Chun-Jun Guo)

**Configuration methods and formulas for key solution reagents and medium**

## **1. Formula of TSAB (Tryptic Soy Agar + blood) plates**

| **Component** | **Amount (in 1 L)** | **Final conc.** | **Vendor (cat. #) [if any]** |
| --- | --- | --- | --- |
| Tryptic Soy Agar | 40 g | 4% (w/v) | BD (236950) |
| Horse blood | 50 mL | 5% (v/v) | Fisher Scientific (50863761) |
| L-Cysteine hydrochloride  50 mg/mL, pH 2.0, in H_2_O | 10 mL | 1% (v/v) | Sigma (C1276) |
| Vitamin K3 1 mg/mL, in EtOH | 1 mL | 0.1% (v/v) |  |

40 g of Tryptic Soy Agar is suspended in 1 L water and autoclaved at 121 °C for 15 min. After cooling the medium down till the temperature is under 60 °C, 50 mL of horse blood, 10 mL of 50 mg/mL L-Cysteine hydrochloride solution and 1 mL of 1 mg/mL Vitamin K3 are added (antibiotics are added at this stage if needed), then the medium is poured into sterile 100 mm x 15 mm Petri Dishes and stock at 4 °C cold room for further use.

## **2. Formula of BHIB (Brain Heart Infusion Agar + blood) plates**

| **Component** | **Amount (in 1 L)** | **Final conc.** | **Vendor (cat. #) [if any]** |
| --- | --- | --- | --- |
| Brain Heart Infusion Agar | 52 g | 5.2% (w/v) | BD (241830) |
| Horse blood | 50 mL | 5% (v/v) | Fisher Scientific (50863761) |
| L-Cysteine hydrochloride  50 mg/mL, pH 2.0, in H_2_O | 10 mL | 1% (v/v) | Sigma (C1276) |
| Vitamin K3  1 mg/mL, in EtOH | 1 mL | 0.1% (v/v) |  |

52 g of Brain Heart Infusion Agar is suspended in 1 L water and autoclaved at 121 °C for 15 min. After cooling the medium down till the temperature is under 60 °C, 50 mL of horse blood, 10 mL of 50 mg/mL L-Cysteine hydrochloride solution and 1 mL of 1 mg/mL Vitamin K3 are added (antibiotics are added at this stage if needed), then the medium is poured into sterile 100 mm x 15 mm Petri Dishes and stock at 4 °C cold room for further use.

## **3. Formula of CBA (Columbia Blood Agar) plates**

| **Component** | **Amount (in 1 L)** | **Final conc.** | **Vendor (cat. #) [if any]** |
| --- | --- | --- | --- |
| Columbia agar | 44 g | 5.2% (w/v) | BD (279240) |
| Horse blood | 50 mL | 5% (v/v) | Fisher Scientific (50863761) |
| L-Cysteine hydrochloride  50 mg/mL, pH 2.0, in H_2_O | 10 mL | 1% (v/v) | Sigma (C1276) |
| Vitamin K3  1 mg/mL, in EtOH | 1 mL | 0.1% (v/v) |  |

44 g of Columbia agar is suspended in 1 L water and autoclaved at 121 °C for 15 min. After cooling the medium down till the temperature is under 60 °C, 50 mL of horse blood, 10 mL of 50 mg/mL L-Cysteine hydrochloride solution and 1 mL of 1 mg/mL Vitamin K3 are added (antibiotics are added at this stage if needed), then the medium is poured into sterile 100 mm x 15 mm Petri Dishes and stock at 4 °C cold room for further use.

## **4. Configuration methods for Mega liquid medium**

| **Component** | **Amount (in 500 mL)** | **Final conc.** | **Vendor (cat. #) [if any]** |
| --- | --- | --- | --- |
| TrypticaseTM Peptone | 5 g | 1% (w/v) | BD (211921) |
| Yeast Extract | 2.5 g | 0.5% (w/v) | BD (212750) |
| D-(+)-Glucose | 1 g | 0.2% (w/v) | Sigma (G8270) |
| L-Cysteine hydrochloride | 0.25 g | 0.05% (w/v) | Sigma (C1276) |
| 1 M Potassium phosphate buffer, pH 7.4^†^ | 50 mL | 10% (v/v) |  |
| TYG salts solution^†^ | 20 mL | 4% (v/v) |  |
| Vitamin K solution^†^ | 500 µL of 1 mg/mL | 0.000001% (w/v) | Sigma (M5625) |
| 0.8% (w/v) CaCl_2_^†^ | 500 µL |  |  |
| FeSO_4_·7H_2_O^†^ | 500 µL of 0.4 mg/mL |  |  |
| Resazurin | 2 mL of 0.25 mg/mL | 0.000001% (w/v) | Sigma (R2127) |
| Histidine-Hematin^†^ | 500 µL |  |  |
| Milli-Q water (dH_2_O) | 150 mL |  |  |
|  |  |  |  |
| † = See “Stock solution recipes” below for details on preparing this component | | | |
|  |  |  |  |
| Next, carbohydrate supplements are added: | | | |
|  |  |  |  |
| **Component** | **Amount (in 500 mL)** | **Final conc.** | **Vendor (cat. #) [if any]** |
| D-(+)-Cellobiose | 0.5 g | 0.1% (w/v) | Sigma (C7252) |
| D-(+)-Maltose monohydrate | 0.5 g | 0.1% (w/v) | Sigma (M5885) |
| D-(-)-Fructose | 0.5 g | 0.1% (w/v) | Sigma (F0127) |
| Soluble starch | 12.5 mL of 2% (w/v) | 0.05% (w/v) |  |
|  |  |  |  |
| Then add: | | | |
|  |  |  |  |
| **Component** | **Amount (in 500 mL)** | **Final conc.** | **Vendor (cat. #) [if any]** |
| Tween 80 | 1 mL of 25% (v/v) | 0.05% (v/v) |  |
| Meat extract | 2.5 g | 0.5% (w/v) | Sigma (70164) |
| Trace Mineral Supplement | 5 mL | 1% (v/v) | ATCC (MD-TMS) |
| Vitamin Supplement | 5 mL | 1% (v/v) | ATCC (MD-VS) |
| SCFA supplement^†^ | 1.4 mL | 0.28% (v/v) |  |
|  |  |  |  |
| † = See “Stock solution recipes” below for details on preparing this component | | | |
|  |  |  |  |
| Adjust pH to pH 7 by and filter-sterilize. Store protected from light at 4 °C (wrap in foil). | | | |
|  |  |  |  |
| **STOCK SOLUTION RECIPES**  **1 M potassium phosphate buffer, pH 7.4** 1. Prepare 1 M KH_2_PO_4_ (monobasic).  • 68.045 g KH_2_PO_4_ (anhydrous, f.w. = 136.09) in Milli-Q water to 500 mL 2. Prepare 1 M K_2_HPO_4_ (dibasic).  • 174.18 g K_2_HPO_4_ (anhydrous, f.w. = 174.18) in Milli-Q water to 1 L 3. Add monobasic to dibasic to achieve pH 7.4.  (You typically need ~430 mL monobasic added to 1 L dibasic)  **Vitamin K solution**  • Dissolve 40 mg menadione (Vitamin K3, Sigma M5625) in 40 mL 100% EtOH.  **TYG salts solution**  • MgSO_4_·7H_2_O (Sigma 230391) 0.5 g  • NaHCO_3_ (Sigma S5761) 10.0 g  • NaCl (Sigma S7653) 2.0 g  • Milli-Q water to 1 L  **FeSO_4_·7H_2_O (0.4 mg/mL)**  • Dissolve 40 mg FeSO_4_·7H_2_O (Sigma F8633) in 100 mL Milli-Q water.  **0.8% (w/v) CaCl_2_**  • Dissolve 0.4 g CaCl_2_·2H_2_O (Sigma C7902) in 50 mL Milli-Q water.  **Resazurin anaerobic indicator (0.25 mg/mL)** 1. Dissolve 25 mg resazurin (Sigma R2127) in 100 mL distilled H_2_O. 2. Store protected from light at 4 °C.  **Histidine-Hematin** 1. Prepare 0.2 M histidine, pH 8.0  • Mix 4.2 g Histidine-HCl monohydrate (Sigma H7875) in 80 mL Milli-Q water.  • Adjust the pH from 4 to 8 with 10 N NaOH (the histidine will go into solution as the pH rises).  • Bring the final volume to 100 mL with Milli-Q water. 2. Mix 12 mg hematin (Sigma H3281) with 10 mL of 0.2 M histidine, pH 8.0. Dissolve by end-over-end rotation or vigorous shaking for several hours. Filter-sterilize using 0.2 µm filter.  **SCFA supplement**  Acetic acid, glacial (Sigma A6283) 17 mL  Propionic acid (Sigma P5561) 6 mL  Butyric acid (Sigma B103500) 4 mL  Isovaleric acid (Sigma 129542) 1 mL | | | |
|  |  |  |  |
|  |  |  |  |
|  |  |  |  |
|  |  |  |  |
|  |  |  |  |
|  |  |  |  |
|  |  |  |  |
|  |  |  |  |
|  |  |  |  |
|  |  |  |  |
|  |  |  |  |
|  |  |  |  |
|  |  |  |  |
|  |  |  |  |
|  |  |  |  |
|  |  |  |  |
|  |  |  |  |
|  |  |  |  |
|  |  |  |  |
|  |  |  |  |
|  |  |  |  |
|  |  |  |  |
|  |  |  |  |
|  |  |  |  |
|  |  |  |  |
|  |  |  |  |
|  |  |  |  |
|  |  |  |  |
|  |  |  |  |
|  |  |  |  |
|  |  |  |  |

## **5. Configuration methods for Chopped Meat Medium (CMM)**

| Ground beef (fat-free) 500.0 g  Distilled water 1.0 L  1N NaOH 25.0 mL  Mix meat, water and NaOH and bring to a boil with stirring. Cool to room temperature, skim fat from surface, and filter, retaining both meat particles and filtrate. To filtrate, add sufficient distilled water to restore volume to 1.0 L.   To this filtrate, add:   Trypticase Peptone (BD 211921) 30.0 g  Yeast extract 5.0 g  K_2_HPO_4_ 5.0 g  0.025% Resazurin 4.0 mL  Boil and cool medium and add:   L-Cysteine hydrochloride 0.5 g  Hemin Solution (see below) 10.0 mL Vitamin K1 Solution (see below) 0.2 mL  Adjust medium for final pH 7.0. Dispense liquid into bottles containing meat particles.   **Vitamin K1 Solution**:  Vitamin K1 0.15 mL 95% Ethanol 30.0 mL  Store solution in brown bottle under refrigeration. Discard after one month.   **Hemin Solution**:  Hemin 50.0 mg  1N NaOH 1.0 mL Distilled water to 100.0 mL  Autoclave at 121 °C for 15 minutes. w/ 15% Sterilized Rumen Fluid from Bar Diamond Ranch (or clarified by centrifugation if prefer)  add 1:100 of 100X carbohydrate stock Cellobiose 0.5 g  Maltose 0.5 g  Fructose 0.5 g Dissolve in 5 mL water, sterile filter |
| --- |
|  |
|  |
|  |
|  |
|  |
|  |
|  |
|  |
|  |
|  |
|  |
|  |
|  |
|  |
|  |
|  |
|  |
|  |
|  |
|  |
|  |
|  |
|  |
|  |
|  |
|  |
|  |
|  |
|  |
|  |

## **6. Configuration methods for the diagnostic PCR reaction in mixed-conjugation strategy**

For Group I conjugation using *E. coli* containing plasmids pGM-ABCM, BBCM, CBCM

| **Component** | **Amount (in 15 µL)** | **Final conc.** | **Vendor (cat. #) [if any]** |
| --- | --- | --- | --- |
| Blue sapphire DNA polymerase | 7.5 µL | 2 times dilution | TAKARA (RR350) |
| pMTL_laz_diag_F | 0.75 µL | 0.5 µM | synthesized from IDT |
| pGM-ABCM_rep_R_1500bp | 0.3 µL | 0.2 µM | synthesized from IDT |
| pGM-BBCM_rep_R_1000bp | 0.3 µL | 0.2 µM | synthesized from IDT |
| pGM-CBCM_rep_R_2000bp | 0.3 µL | 0.2 µM | synthesized from IDT |
| Genomic DNA | 1 µL |  |  |
| H_2_O | 4.85 µL |  |  |

For Group II conjugation using *E. coli* containing plasmids pGM-DBCM, EBCM, FBCM

| **Component** | **Amount (in 15 µL)** | **Final conc.** | **Vendor (cat. #) [if any]** |
| --- | --- | --- | --- |
| Blue sapphire DNA polymerase | 7.5 µL | 2 times dilution | TAKARA (RR350) |
| pMTL_laz_diag_F | 0.75 µL | 0.5 µM | synthesized from IDT |
| pGM-DBCM_rep_R_1000bp | 0.3 µL | 0.2 µM | synthesized from IDT |
| pGM-EBCM_rep-MU102_R_1500 | 0.3 µL | 0.2 µM | synthesized from IDT |
| pGM-FBCM_rep-AMβ1_R_2000 | 0.3 µL | 0.2 µM | synthesized from IDT |
| Genomic DNA | 1 µL |  |  |
| H_2_O | 4.85 µL |  |  |

For Group III conjugation using *E. coli* containing plasmids pGM-GBCM, HBCM, IBCM

| **Component** | **Amount (in 15 µL)** | **Final conc.** | **Vendor (cat. #) [if any]** |
| --- | --- | --- | --- |
| Blue sapphire DNA polymerase | 7.5 µL | 2 times dilution | TAKARA (RR350) |
| pMTL_laz_diag_F | 0.75 µL | 0.5 µM | synthesized from IDT |
| pGM-GBCM_rep-pWV01_R_1000 | 0.3 µL | 0.2 µM | synthesized from IDT |
| pGM-HBCM_rep-pMB1_R_1500 | 0.3 µL | 0.2 µM | synthesized from IDT |
| pGM-IBCM_rep_R_2000 | 0.3 µL | 0.2 µM | synthesized from IDT |
| Genomic DNA | 1 µL |  |  |
| H_2_O | 4.85 µL |  |  |

*Note*: IDT, Integrated DNA Technologies.
